# 

**Published:** 1857-01

**Authors:** 


### LIST OF COLLABORATORS.

---

Walter F. Atlee, M.D., Philadelphia.

John Bell, M.D., Philadelphia, formerly Professor of Medicine, Medical College of Ohio.

T. S. Bell, M.D., Louisville, Ky.

S. M. Bemiss, M.D., Louisville, Ky.

L. P. Blackburn, M.D., Natchez, Miss.

G. C. Blackman, M.D., Professor of Surgery, Medical College of Ohio.

J. L. Bobbs, M.D., formerly Professor of Anatomy, Indianapolis.

W. M. Boling, M.D., Montgomery, Ala., formerly Professor of Midwifery, Transylvania University, Lexington, Ky.

N. Bozeman, M.D., Montgomery, Ala.

J. T. Bradford, M.D., Augusta, Ky.

John H. Brinton, M.D., Lecturer on Operative Surgery, Philadelphia.

W. S. Chipley, M.D., Professor of Medicine, Transylvania University.

A. Clapp, M.D., New Albany, Ia.

D. B. Cliffe, M.D., Franklin, Tenn.

J. Da Costa, M.D., Lecturer on the Practice of Medicine, at the Philad. Med. Association.

M. Dupierris, M.D., Havana, Cuba.

W. F. Edgar, M.D., United States Army.

S. C. Farrar, M.D., Jackson, Miss.

C. S. Fenner, M.D., Memphis, Tenn.

Austin Flint, M.D., Professor of Clinical Medicine and Pathology, University of Buffalo.

W. Y. Gadbury, M.D., Benton, Miss.

O. C. Gibbs, M.D., Chautauque County, New York.

Dr. J. P. Hall, Glasgow, Ky.

John Hardin, M.D., Professor of Obstetrics, Kentucky School of Medicine, Louisville.

Edward Hartshorne, M.D., One of the Surgeons to Wills' Hospital for Diseases of the Eye, Philadelphia.

E. B. Haskins, M.D., Clarksville, Tenn., late President of the Tennessee State Medical Society.

C. A. Hentz, M.D., Marianna, Florida.

ADDINELL HEWSON, M.D., One of the Surgeons to Wills' Hospital for Diseases of the Eye, Philadelphia.

SAMUEL L. HOLLINGSWORTH, M.D., late Editor of the Medical Examiner, Philadelphia.

WILSON JEWELL, M.D., Philadelphia.

G. A. KUNKLER, M.D., Madison, Ia.

BEN. LOGAN, M.D., Shreveport, La.

A. LOPEZ, M.D., Mobile, Ala., formerly President of the Alabama State Medical Society.

B. I. RAPHAEL, M.D., New York.

J. C. REEVE, M.D., Dayton, Ohio.

L. C. RIVES, M.D., formerly Professor of Midwifery, Medical College of Ohio.

J. LAWRENCE SMITH, M.D., Professor of Chemistry, University of Louisville.

W. C. SNEED, M.D., Frankfort, Ky., President, Kentucky State Medical Society.

C. H. SPILMAN, M.D., Harrodsburg, Ky., late President of Kentucky State Medical Society.

GEO. SUTTON, M.D., Aurora, Ia.

W. L. SUTTON, M.D., Georgetown, Ky., formerly President Kentucky State Medical Society.

S. THOMPSON, M.D., Albion, Ill.

E. WILLIAMS, M.D., Cincinnati, Ohio.

### Bi-Monthly Bibliographical Record.

### AMERICAN.

Chemical and Pharmaceutical Manipulations. By Campbell Morfit, Professor of Chemistry, University of Maryland, and Clarence Morfit, Assistant Melter and Refiner in the United States Assay Office. Second Edition. With five hundred and thirty-seven illustrations. 8vo. pp. 626. Lindsay & Blakiston, Philadelphia, 1856. (Received from the Publishers.)

Report of a Case of Hydrophobia. From Dr. Blatchford's Report to the American Medical Association. By W. L. Atlee, M.D. Pamphlet, pp. 38. Philadelphia, 1856. (Received from the author.)

The Transactions of the American Medical Association. Vol. IX. 8vo. pp. 907. Philadelphia, 1856. (Received from the Publishing Committee.)

The Therapeutical Powers and Properties of Veratrum Viride. By W. C. Norwood, M.D., of Cokesbury, S. C. Second Edition. Pamphlet; pp. 23. J. D. Bedford & Co., New York, 1856. (Received from the Author.)

On the Treatment of Iritis without Mercury. By H. W. Williams, M.D., one of the Surgeons to the Boston Dispensary. Pamphlet; pp. 24. Reprinted from the Boston Medical and Surgical Journal. David Clapp, Boston, 1856. (Received from the Author.)

The Practical Anatomist. By J. M. Allen, M.D., late Professor of Anatomy, Penn. Med. College. 8vo., pp. 631. Blanchard & Lea, Philadelphia, 1856. (Received from the Publishers.)

Obstetrics; the Science and the Art. By Chas. D. Meigs, M.D., Professor of Midwifery and the Diseases of Women and Children, in the Jefferson Medical College, at Philadelphia, &c. &c. Third Edition, revised; with 120 illustrations. 8vo., pp. 758. Blanchard & Lea, Philadelphia, 1856. (Received from the Publishers.)

The History, Diagnosis, and Treatment of the Fevers of the United States. By Elisha Bartlett, M.D., late Professor of Materia Medica and Medical Jurisprudence, in the College of Physicians and Surgeons, New York. Fourth edition; revised by A. Clark, M.D., &c. 8vo., pp. 604. Blanchard & Lea, Philadelphia, 1856. (Received from the Publishers.)

### FOREIGN.

Medical Notes and Reflections. By Sir Henry Holland, Bart., F.R.S., &c. &c. First American, from the third London edition. 8vo., pp. 494. Blanchard & Lea, Philadelphia, 1856. (Received from the Publishers.)

Hand-Book of Organic and Inorganic Chemistry. By Wm. Gregory, M.D., Professor of Chemistry, University of Edinburgh. Fourth American, from the third English edition. Edited by J. M. Sanders, M.D., Professor of Chemistry, Eclectic Medical Institute, Cincinnati. 2 vols., 8vo., pp. 896. A. S. Barnes & Co., New York. (Received from the Publishers.)

An Introduction to Practical Chemistry, including Analysis. By John E. Bowman, F.C.S. Second American, from the second London edition. pp. 298. Blanchard & Lea, Philadelphia, 1856. (Received from the Publishers.)

Lectures on the Principles and Methods of Medical Observation and Research, for the Use of advanced Students and Practitioners. By Thomas Laycock, M.D., Professor of the Practice of Medicine, University of Edinburgh. 1856.

The Dissector's Manual of Practical and Surgical Anatomy. By Erasmus Wilson, F.R.S. Third American, from the last London edition. Edited by Wm. Hunt, M.D., Demonstrator of Anatomy, University of Pennsylvania. 8vo., pp. 580. Blanchard & Lea, Philadelphia, 1856. (Received from the Publishers.)

The Physician's Prescription Book; containing List of Terms, Phrases, &c., used in Prescriptions, with Explanatory Notes; also the Grammatical Construction of Prescriptions, &c. &c. By Jonathan Pereira, M.D., F.R.S. Second American, from the twelfth London edition. 12mo., pp. 282. Lindsay & Blakiston, Philadelphia, 1857. (Received from the Publishers.)

Practical Anatomy. A new Arrangement of the London Dissector. By D. H. Agnew, M.D., Lecturer on Anatomy, &c. Pp. 310. Blanchard & Lea, Philadelphia, 1856. (Received from the Publishers.)

Des Aneurysms et de leur Traitement. Par P. Broca, Agrége a la Faculté de Médecine de Paris, Chirurgeon des Hôpitaux, &c. 8vo., pp. 931. Paris, 1856. (Received from the Author.)

